# Applications of artificial intelligence in orthopaedic surgery

**DOI:** 10.3389/fmedt.2022.995526

**Published:** 2022-12-15

**Authors:** Faraz Farhadi, Matthew R. Barnes, Harun R. Sugito, Jessica M. Sin, Eric R. Henderson, Joshua J. Levy

**Affiliations:** ^1^ Geisel School of Medicine, Dartmouth College, Hanover, NH, United States; ^2^ Radiology and Imaging Sciences, National Institutes of Health (NIH), Bethesda, United States; ^3^ Department of Radiology, Dartmouth Health, Lebanon, United States; ^4^ Department of Orthopaedics, Dartmouth Health, Lebanon, United States; ^5^ Department of Pathology and Laboratory Medicine, Dartmouth Health, Lebanon, NH, United States

**Keywords:** artificial intelligence, Orthopaedics, Orthopaedic Surgery, machine learning, deep learning

## Abstract

The practice of medicine is rapidly transforming as a result of technological breakthroughs. Artificial intelligence (AI) systems are becoming more and more relevant in medicine and orthopaedic surgery as a result of the nearly exponential growth in computer processing power, cloud based computing, and development, and refining of medical-task specific software algorithms. Because of the extensive role of technologies such as medical imaging that bring high sensitivity, specificity, and positive/negative prognostic value to management of orthopaedic disorders, the field is particularly ripe for the application of machine-based integration of imaging studies, among other applications. Through this review, we seek to promote awareness in the orthopaedics community of the current accomplishments and projected uses of AI and ML as described in the literature. We summarize the current state of the art in the use of ML and AI in five key orthopaedic disciplines: joint reconstruction, spine, orthopaedic oncology, trauma, and sports medicine.

## Introduction

**Figure 1:**
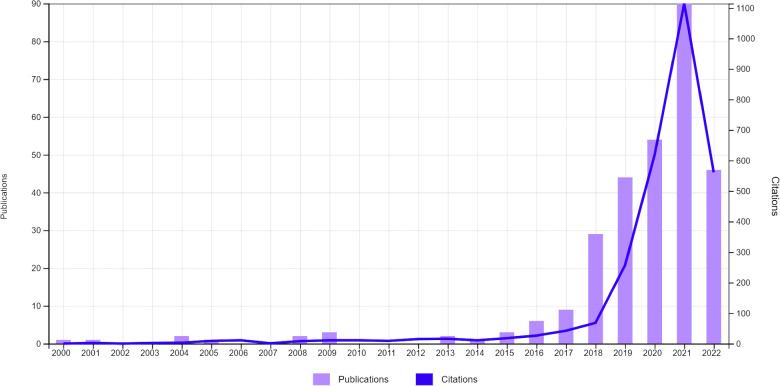
Evolution of the number of total publications and citations whose title, abstract and/or keywords refer to the field of artificial intelligence in orthopaedics during the last years. Data retrieved from Web-of-science (June 20th, 2022) by using the search term: orthopaedic* AND (Artificial Intelligence OR Deep Learning OR Machine Learning OR Convolutional Neural Network).

Orthopaedic surgery is the field of medicine dedicated to addressing–through invasive and non-invasive strategies–traumatic and pathological processes affecting the bones, joints, and adjacent connective tissues facilitating motor function. Because the skeleton, and subtle skeletal pathology, projects well on photon attenuation-based radiography and computed tomography (CT), and because soft-tissue injuries are diagnosed with high sensitivity, specificity, and positive/negative prognostic value tools such as magnetic resonance imaging (MRI), orthopaedic surgery is a field that is particularly ripe for the application of machine-based integration of imaging studies, among other applications. Although the use of computer algorithms to diagnose and manage orthopaedic disorders dates back to the 20th century, the literature shows that interest in this area among medical scientists has markedly increased during the past decade (Figure 1). Improvements in software and hardware technologies, such as graphic processing units and cloud computing, has allowed for substantially faster processing computers and storage of larger amounts of data, resulting in development of machines increasingly capable of performing tasks that typically require human intelligence.

Artificial intelligence (AI) is neither a new discipline nor is its application to the medical field a new phenomenon. Before the field was born, many works were pursued that were later recognized as AI, including statistics-based methods, such as logistic regression (LR) (1). The term was first coined by John McCarthy in a proposal to study the concept during a research event in the summer of 1956 at Dartmouth College. However the idea of intelligence in machines was investigated prior to this event including in the works of Alan Turing who raised the question of “can a machine think?” McCarthy and colleagues submitted their proposal on the assumption that every feature of learning or any other trait of intelligence can be characterized precisely enough for a computer to emulate it (2).”

**Figure 2:**
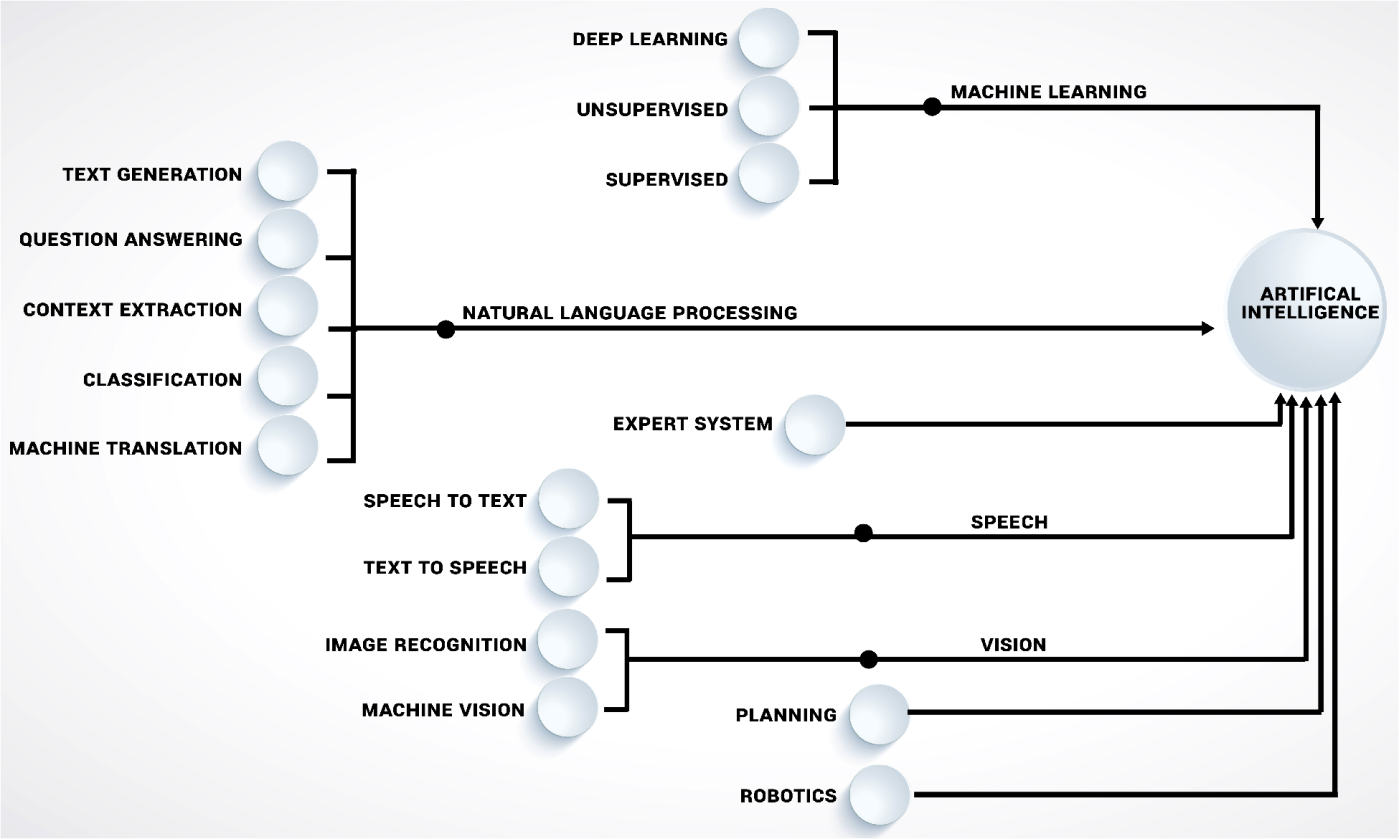
Illustrating the key disciplines of artificial intelligence (AI), including machine learning (ML) approaches that are influencing orthopaedics research.

Today, the field of AI is composed of many sub-branches (Figure 2), with a particular focus of this study being machine learning (ML). While other AI branches have had a significant influence on science and technology, ML is without a doubt the most intriguing and promising discipline of AI for medical research applications today. Even though AI and ML are sometimes used interchangeably in the public, ML more accurately described as a subset of AI that uses algorithms with the capacity to “learn.” The overall objective of machine learning is for computers to adapt without being instructed on how, in order to predict the value of a desired output based on a set of inputs. Enabling computers to learn how to perform tasks on their own, with or without human guidance on what to do and what not to do (i.e., supervised vs. unsupervised ML), often in ways that exceeds human understanding, opens up many potential use cases. While both supervised and unsupervised machine learning techniques have been widely used in orthopaedic, each has advantages and disadvantages. Algorithms in supervised learning are guided by human-defined ground truth and compared to unsupervised learning, they are often easier to implement and have a more understandable decision-making process. However, creating high quality human-defined ground truth necessitates a significant manual effort and also incfeases risk of incorporating human biases into decision making. Unsupervised learning, as opposed to supervised learning, derives insights directly from the data, groups the data, and aids in the formulation of data-driven decisions without introducing external bias (3, 4).

**Figure 3:**
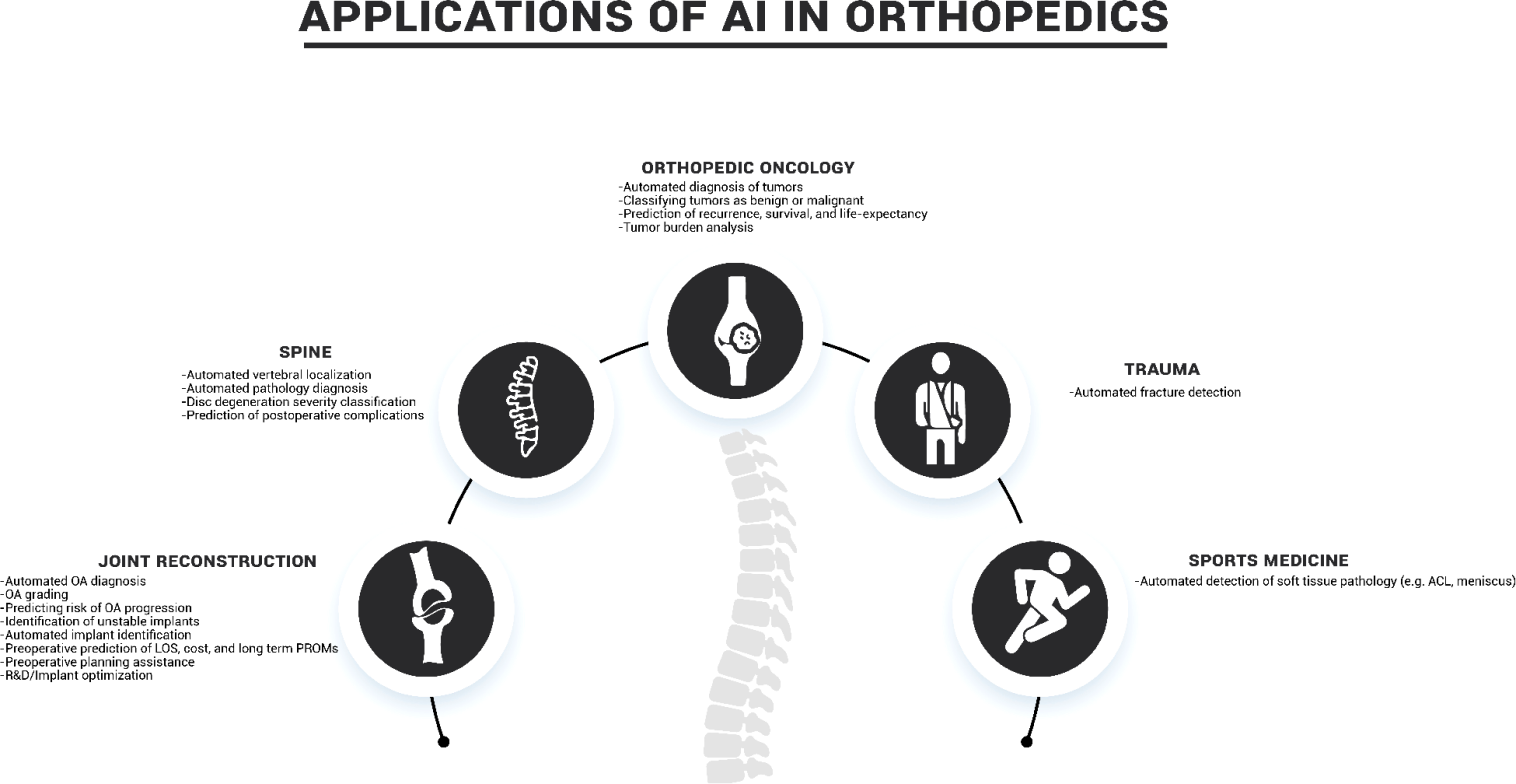
Common applications of AI in orthopaedics.

Through this literature review we seek to promote awareness in the orthopaedics community of the current accomplishments and projected uses of AI and ML as described in the literature (Table 1). The following sections summarize the current state of the art in the use of ML and AI in five key orthopaedic disciplines: joint reconstruction, spine, orthopaedic oncology, trauma, and sports medicine (Figure 3).

**Table 1:** Summarizes selected manuscripts for application of AI in orthopaedics that were discussed in this article, including their purpose, AI technology that was used, their study population and figures of merit.

Author, year	Purpose	AI technology	Study population	FoM
Joint Reconstruction				
Xue et al. 2017	Hip OA diagnosis	VGG-16 Layer CNN	420 hip x-rays	SN: 95.0%, SP: 90.7%, AUC: 0.94
Üreten et al. 2020	Hip OA diagnosis	VGG-16 Layer CNN	434 hip x-rays	SN: 97.6%, SP: 83.0%
Tiulpin et al. 2018	Knee OA diagnosis	Deep Siamese CNN	5,960 knee x-rays	AUC: 0.93, QWK: 0.83, MSE: 0.48, MCA: 66.7%
Swiecicki et al. 2021	Knee OA Severity	VGG-16 Layer CNN	3,200 patients’ knee x-rays and 10,052 exams	QWK: 0.91 MCA: 71.9%
Kim et al. 2020	Knee OA Severity	CUDA/cuCNN	3,000 patients’ knee x-rays and exams	AUC: 0.75-0.97
Ashinski et al. 2017	Early Knee OA Prediction	WND-CHRM	68 subjects with T_2_-weighted Knee MRIs and 3 year follow ups	SN: 74%, SP 76%
Pedoia et al. 2019	Early Knee OA Prediction	DenseNet CNN	4,384 subjects T_2_-weighted Knee MRIs and 3 year follow ups	SN: 77.0%, SP: 77.9%, AUC: 0.824
Borjali et al. 2019	Detection of Implant Loosening	Deep CNN	40 hip x-rays	SN: 94%, SP: 96%, AUC
Yi et al. 2020	Knee Implant Detection and Identification	Deep CNN	511 knee x-rays	SN: 100%, SP: 100%, AUC 1.00 for native vs. TKA vs. UKA and descrimination between implant models
Yi et al. 2020	Shoulder Implant Detection and Identification	Deep CNN	482 shoulder x-rays	AUC: 1.00 for detection of implant; AUC: 0.97 for differentiation between TSA and RTSA; AUC: 0.86-1.00 for discrimination between implant models
Ramkumar et al. 2019	Prediction of Length of Stay and Inpatient Costs for THA	Naive Baysian Model	122,334 THA surgery patients’ records	AUC: 0.87 for LOS AUC 0.71 for payment
Navarro et al. 2018	Prediction of Length of Stay and Inpatient Costs for TKA	Naive Baysian Model	141,446 TKA surgery patients’ records	AUC: 0.78 for LOS AUC 0.74 for payment
Fontana et al. 2019	Prediction of MCID of PROMs after Arthroplasty	Lasso, Random Forest, and SVM	7,239 THA and 6,480 TKA surgeries	AUC: 0.60-0.89
Lambrechts et al. 2022	Improving Presurgical Planning Workflow	Lasso and SVM	5,409 TKA surgeries	39.7% improvement in number of corrections to presurgical plan
Eskinazi et al. 2015	Implant Optimization	ANN		∼1000× faster computational time; ∼7× more accurate
Cilla et al. 2017	Implant Optimization	ANN and SVM		Decreased strain in optimized implant, SVM showed higher accuracy
**Spine**				
Hetherington et.al., 2017	Automatic spine level identification	Deep learning- AlexNet, GoogLeNet, ResNet-50, SqueezeNet	Ultrasound images of L1-S1 vertebral bodies from 20 participants	AC: 88-91% between different algorithms
Glocker et al. 2012	Automatic localization and identification of vertebrae	Deep learning- random forest	200 CT images	Localization error: 6-8.5 mm Identification rate: 81%
Chen et al. 2015	Automatic localization and identification of vertebrae	Deep learning- random forest and CNN	MICCAI 2014 vertebral localization challenge: 302 annotated spine CT with post-op and pathologic cases	Localization error: 1.6-2 mm
Bounds et al. 1988	classifying back pain in four categories of simple, radicular, pathologic, and back pain with significant psychological overlay	Multilayer perceptron network	Clinical symptoms and past medical history of 200 patients with back pain	AC: 77-82%
Ghosh et al. 2011	Image based diagnosis of lumbar herniation	five different classifiers including SVM	35 lumbar MRI	AC: 80-94% between different classifiers
Hao et al. 2013	Image based diagnosis of lumbar herniation	Deep learning- SVM	MRI of 162 disks from 27 patients	AC: 92%
Oncology				
Han et al. 2018	To predict survival rates of patients with synovial sarcoma	Deep learning - Neural Network	Clinical and demographic data - 242 patients from 3 institutions	ROC AUC: 0.81
He et al. 2020	To differentiate benign vs intermediate vs malignant primary bone tumors	Deep learning -efficientNet-B0 CNN	1356 Plain radiographs from 5 institutions	AC:73.4% on external validation
Eweje et al. 2021	To differentiate benign vs malignant primary bone tumors	Deep learning -efficientNet-B0 CNN	T1- and T2- weighted MRI - 1,060 lesions - 4 institutions	AC:73% ROC AUC: 0.82
He et al. 2019	To predict local recurrence of giant cell tumor of bone after intralesional curettage	Deep learning- Inception v3, LR	MRI, age, tumor location - 56 patients	AC: 75.5%, SN: 85.7%
Zhang et al. 2018	Segmentation of osteosarcoma on CT images	Deep learning- multiple supervised residual network	CT images - 23 patients	DSC: 89.2%
Lindgren et al. 2019	Automatic segmentation for tumor burden calculation from prostate cancer bony metastasis	Deep learning- CNN	18F-sodium fluoride PET/CT - 48 patients	70% concordance index for prediction of overall survival
**Trauma**				
Author, year	Purpose	AI technology	Study population	FoM
Olczak et al. 2017	Fracture detection	Deep learning networks	256,000 wrist, hand, and ankle radiographs	>90% accuracy for identifying laterality, body part, and view 83% accuracy for fracture detection
Adams et al. 2019	Neck of femur fracture detection	GoogLeNet and AlexNet Deep CNN	805 hip radiographs	Accuracy: 85.3-90.6%, AUC: 0.89-0.98
Urakawa et al. 2019	Intertrochanteric hip fracture detection	VGG-16 CNN	3,346 hip radiographs	Accuracy: 95.5 SN: 93.9%, SP: 97.4%
Gan et al. 2019	Distal radius fracture detection	Faster R-CNN and Inception-v4 CNN	2,340 AP wrist radiographs	AUC: 0.96
Chung et al. 2018	Proximal humerus fracture detection	ResNet-152 deep CNN	1,891 shoulder radiographs	Accuracy: 96%, AUC: 0.97-1.00 for fracture detection Accuracy: 65-86%, AUC: 0.90-0.98 for classification by fracture type
Pranata et al. 2019	Calcaneus fracture detection	ResNet and VGG deep CNN	1,931 foot CT images	Accuracy: 98%
Krogue et al 2020	Hip fracture detection	DenseNet CNN	1,118 hip and pelvic radiographs	Accuracy: 93.7%, SN: 93.2%, SP: 94.2%. Improvement in physician performance when aided
Lindsey et al. 2018	Fracture detection	Deep convolutional neural network (DCNN)	135,845 radiographs of a variety of body parts (wrist,foot, elbow, shoulder, knee, spine, femur, ankle, humerus, pelvis, hip, and tibia)	AUC: 0.990 SN: 93.9%, SP: 94.5%. Improvement in emergency medicine MD SN from 82.7% to 94.5%, SP from 87.4% to 94.1%.
Karnuta et al. 2019	Post-operative length of stay and cost prediction for hip fracture	Bayes machine-learning algorithm	98,562 Medicare patients who underwent operative management for hip fracture	Accuracy: 76.5% for length of stay and 79.0% for cost. AUC: 0.88 for length of stay and 0.89 for cost.
Stojadinovic et al. 2011	Fracture non-union prediction	Bayesian belief network	349 patients with delayed fracture union or a nonunion	Mean AUC: 0.66, PPV: 0.86, NPV: 0.29
**Sports Medicine**				
Štajduhar et al. 2017	Anterior cruciate ligament (ACL) injury detection	Support vector machine (SVM) and random forests models	969 sagittal knee MRs	AUC: 0.894 for injury detection and 0.943 complete-rupture detection
Bien et al. 2018	ACL and meniscal tears detection	MRNet CNN	1,370 knee MRIs	AUC: 0.937, SN: 0.879, SP: 0.714, Accuracy: 0.850 for abnormality detection
Chang et al. 2019	Complete ACL tear detection	ResNet-derived CNN	Knee MRIs from 260 patients	AUC: 0.971, SN: 0.967, SP: 1.00, PPV: 0.938, NPV: 1.0
Roblot et al. 2019	Meniscus tear detection	Fast-region CNN (RCNN) and faster-RCNN	1,123 MR images of the knee	AUC: 0.90

FoM, figures of merit; AC, accuracy;ROC, receiver operator characteristic; AUC, area under the curve; SN, sensitivity; SP, specificity; DSC, dice similarity coefficient; QWK, quadratic weighted Kappa; MSE, mean square error; MCA, multi-class accuracy.

## Joint reconstruction

As joint reconstruction is one of the major orthopaedics subspecialties, it comes as no surprise that this has also been a key area of studies for AI within orthopaedics. Common use cases for AI in joint reconstruction have been analyzing imaging for automated diagnosis, evaluation of implants, and predicting clinical outcomes, as well as more niche examples such as streamlining pre-operative workflow for patient specific implants and implant R&D.

### Automated image-based diagnosis

Osteoarthritis (OA) is the most common joint disorder in the US, affecting 10% of men and 13% of women over the age of 65 (5). Given this high prevalence, a major area of focus for ML in the field of orthopaedics has been automating detection and staging of OA from imaging studies. Xue et al. and Üreten et al. used a VGG-16 layer deep convolutional neural network (CNN) to automatically diagnose hip OA from radiographs (6, 7). The Xue et al. model achieved a high sensitivity of 95% and specificity of 90.7%, comparable to an experienced physician. Similarly, Tiulpin et al. used a Deep Siamese CNN to automatically diagnose and grade knee OA from radiographs (8). This model also helps to open up the “black box” of AI by specifically highlighting the key radiologic features that determine the diagnosis to help build trust with the physician user.

In addition to making the diagnosis of OA from radiographs, several studies have utilized AI to further categorize OA severity. Swiecicki et al. used a Faster R-CNN model on radiographs to assess severity of knee OA based on the Kellgren-Lawrence grading system (9). Their model had a similar accuracy to a panel of radiologists, with improved reproducibility. Kim et al. performed a similar study using deep learning and found significantly improved areas under the curve (AUCs) when combining the image data with information about patient demographics and medical history (10).

While radiographs are the primary diagnostic tool to evaluate arthritis, several studies have used MRI to provide more detailed information. Ashinsky et al. used the machine learning tool weighted neighbor distance using compound hierarchy of algorithms representing morphology (WND-CHRM) to evaluate T_2_ weighted MRI sequences to identify medial femoral condyles at risk for progression to symptomatic OA, as defined by a change in Western Ontario and McMaster Universities Arthritis (WOMAC) score of >10 within three years (11). This model achieved sensitivity of 74% and specificity of 76%, though was limited in sample size and only evaluated a single compartment. A more generalized model of the whole knee was created by Pedoia et al. using DenseNet, a densely connected CNN, which used T_2_ weighted MRI sequences to diagnose knee OA prior to visible radiographic changes (12). When combined with patient demographics of age, gender, BMI, and Knee injury and Osteoarthritis Outcome Score (KOOS), their model demonstrated sensitivity of 76.99% and specificity of 77.94% among several thousand patients in the Osteoarthritis Initiative baseline dataset.

### Automated implant evaluation

One major cause of failure of arthroplasty implants is mechanical loosening, which can be caused by inadequate initial fixation, loss of fixation over time, biological loss of fixation due to osteolysis, and/or periprosthetic infection (13, 14). Following joint replacement, it is critical to evaluate implant imagingfor hardware complications. Borjali et al. trained a deep CNN to detect mechanical loosening of total hip implants from radiographs (15). This model achieved significantly higher sensitivity of 94% and similar specificity of 96% as compared to an experienced orthopaedic surgeon. They also created outputs of saliency maps showing the key areas the model used to make the diagnosis, helping to build trust in the results.

In addition, if revision becomes necessary, identifing the specific implant used in the primary surgery is a key step in preoperative planning, however, roughly 10% of implants are unable to be identified preoperatively (16). Yi et al. addressed this issue with a ResNet deep CNN to characterize knee radiographs based on presence or absence of knee arthroplasty, classification of total vs. unicompartmental knee arthroplasty, and differentiation between two different manufacturers' implant products (17). Yi et al. conducted a similar study for shoulder arthroplasty, discriminating between native, total arthroplasty, and reverse arthroplasty shoulders, as well as differentiating between five different models of implants (18). This particular use case for ML has the potential to offer time savings for surgeons during pre-operative planning of revision cases.

### Clinical outcome prediction

Another area of focus for ML in joint reconstruction has been prediction of surgical outcomes. Given the particular reimbursement challenges in joint replacement with Medicare's Comprehensive Care for Joint Replacement (CJR) bundled reimbursement model, Ramkumar et al. proposed a value-based, patient-specific payment model using pre-operative outcome forecasting in hip and knee arthroplasty (19, 20). They used a Bayesian approach to accurately predict length of stay and cost following total knee and hip arthroplasty based on patient characteristics including age, ethnicity, gender, and comorbidities and proposed tiered patient specific reimbursement to more fairly account for patient complexity as compared to CJR. Examining clinical outcomes, Harris et al. used a Least Absolute Shrinkage and Selection Operator (LASSO) model to make moderately accurate predictions of 30 day mortality and cardiac complications following total joint replacement (21). Fontana et al. focused on prediction of longer term patient reported outcome measures (PROMs) and identification of patients at risk of not achieving meaningful gains to facilitate presurgical decision support (22). They compared three ML algorithms, support vector machine (SVM), LASSO, and random forest, finding they all had similar fair-to-good predictive power in 2 year meaningful gains in PROMs when analyzed before surgery. Tools such as these have the potential to be powerful resources for patients and physicians for medical decision making, as they can avoid bias and heuristics, time constraints of physicians, while offering decision aids, identification of modifiable risk factors, and prognostication of outcomes and complications (23). However, use in real-world scenarios will likely require tools with higher levels of accuracy, as well as addressing issues with interpretability, integration with electronic health records, ongoing monitoring and validation of results, and ethical concerns.

### Improving surgeon workflow

One growing trend in orthopaedic arthroplasty has been the use of patient specific implants, allowing for potentially superior outcomes, though involves more work during pre-operative planning (24). Lambrechts et al. developed a novel application for ML in patient-specific joint replacement by using ML to automate patient- and surgeon-specific preoperative planning (25). Combining LASSO and SVM approaches, the AI-based preoperative plans were significantly improved as compared to the manufacturer's plans by requiring fewer manual corrections by the surgeon, thus streamlining the surgeons' preoperative workflow and reducing time needed to make corrections.

### Implant research and development

Within the orthopaedic medical device industry, the market for arthroplasty implants is generally mature, with manufacturers often struggling to differentiate their products in terms of clinical benefit over competitors. Recently, several studies have examined ML's role in R&D and optimization of arthroplasty implants. Eskinazi et al. streamlined a deformable joint contact model by using a feed-forward artificial neural network (ANN) model to estimate thousands of loading conditions for an artificial knee implant (26). The ANN computed contact forces and torques more accurately and nearly 1,000 times faster than the traditional elastic foundation modeling method, removing a significant hurdle for regular use of this technique in implant optimization. Cilla et al. used two different machine learning techniques, SVM and ANN, in combination with Finite Element to assess their utility in optimizing short stem hip implant geometry (27). Both techniques significantly reduced computational time, with SVM providing a higher degree of accuracy in stress shielding quantification compared to ANN. Further investigation into implant optimization could help to improve the long term performance of orthopaedic implants and potentially further differentiate products on the market.

## Spine

Based on the number of research publications in the field, spine surgery has been another of the major areas of AI research in orthopaedics. Some of the more explored topics include diagnosing spinal pathologies from clinical or imaging information and predicting postoperative complications.

### Automated image localization and labeling

The initial step in developing algorithms for detecting and classifying pathologic abnormalities on imaging is to locate anatomical structures. In orthopaedic spine surgery, due to intricate structural pathologies and anatomical variations in vertebral bodies, localization can also directly help with clinical procedures by enhancing accuracy and speed. Hetherington et al. developed multiple CNN algorithms (AlexNet, GoogLeNet, ResNet-50, SqueezeNet) for real-time identification of the vertebral level on ultrasound images. Authors reported a detection accuracy of 88%–91% between different algorithms for accuracy in classification of vertebral bodies. Translational applications of such algorithms would be beneficial in neuraxial anesthesia and analgesia, including spinal and epidural needle insertions and facet joint injections, in addition to reducing risk of surgery at the wrong spine level (28). This could improve accuracy over methods such as palpation and loss-of-resistance, while reducing radiation exposure over fluoroscopic guided treatments (29).

Localization has also been applied to images with spinal pathologies. Glocker et al. used a classification random forest to determine the location of vertebral centroid on CT images from patients with severe scoliosis, sagittal deformity, and presence of fixation devices. They reported a mean localization error of between 6 and 8.5 mm (30). Chen et al. used a two step method to first localize the center of each intervertebral discs and then segment the discs utilizing a random forest classifier and deep CNN. Their method showed significant improvement to other previous non-deep learning methods with an average localization error for the centroid of the intervertebral disk of 1.6–2 mm (31).

Today, cutting-edge techniques for locating and labeling spinal structures perform as-well-as experienced human observers. In fact, many commercially available picture archiving and communication systems (PACS) and medical imaging software include these features (32).

### Automated image-based diagnosis

Diagnosing spine pathologies has been a major focus of AI research in spine orthopaedics. The use of ML for diagnosing spine pathologies dates back to the 1980s. In a 1988 study, Bounds et al. reported diagnostic accuracy of 77%–82% for training a multilayer perceptron for classifying back pain in four categories of simple, radicular, pathologic (e.g., tumor, infection, inflammation), and back pain with significant psychological overlay based on clinical symptoms and previous medical history (33).

A variety of AI methods have been explored for image-based diagnosis of spine pathologies, such as non-ML approaches derived from traditional image processing techniques, as well as basic ML techniques like Bayesian classifiers (34, 35). Most of these methods use image pixel value (CT Hounsfield Unit or MRI signal intensity) and texture information to train and test diagnostic algorithms. Ghosh et al. used MRI trained on numerous different classifiers, including an SVM, to classify intervertebral discs as degenerated or normal. Accuracy ranged from 80 to 94 percent, with SVM being the most accurate (36). Hao et al. used morphological information, such as the shape of the disc, in addition to image pixel value and texture information to train an SVM-based model to classify discs as degenerated or normal, obtaining accuracies of up to 92% (37).

There are other methods of intervertebral disc classification besides the binary classification of healthy or degenerated. One classification that is clinically used was proposed by Pfirrmann et al. and describes five degeneration degrees based on MRI signal characteristics (38). Castro-Mateos et al. used MRI images of lumbar spine to train and test neural network utility on classification of disc degeneration based on Pfirrmann classification. They reported a mean specificity and sensitivity of 93% and 83% (39).

### Postoperative complication prediction

Developing high-accuracy preoperative prognostication models would enhance patient counseling and shared decision-making through more accurate forecasting of potential adverse events. This could be particularly true in acute settings, such as high-energy trauma, where there is limited time for considering available options. In the past decade many articles have been published investigating models made for predicting various aspects of outcomes of spine surgeries.

McGirt et al. used regression to predict outcomes after lumbar surgery. They used several predictor variables, such as age, BMI, detailed symptoms, and presence of spinal disorders to predict clinical outcomes, including 12-month Oswestry Disability Index (ODI), 30-day readmission rates, rehabilitation needs, and return to work, achieving accuracies between 72% and 84% (40).

Kim et al. trained and validated deep learning models to identify risk factors for postoperative complications of posterior lumbar spine fusion using CNN and LR. They used information from 22,629 patients from the American College of Surgeons National Surgical Quality Improvement Program, including demographic and clinical variables to predict cardiac and wound complications, venous thromboembolism, and mortality. Both CNN and LR showed higher AUC in predicting all four outcome variables when compared to American Society of Anesthesiology classification (41). In a different study, the same group confirmed similar results in a cohort (*n* = 4,073) of patients undergoing elective adult spinal deformity procedures (42).

Scheer et al. used a data set of 657 patients that underwent spine deformity surgery with and without intra- or perioperative complications. Authors trained an ensemble of decision trees to use baseline demographic, radiographic, and surgical factors to predict the possibility of major complications with an overall accuracy of 87.6%. They reported age, leg pain, OSI, ASA grade, presence of osteoporosis, and pelvic tilt among the highest predictor variables (43).

One strategy to incorporate predictive analytics into regular clinical practice is to use a decision support tool, which leverages the predictive capacity of the models to help clinical decisions by providing personalized suggestions. As detailed by Coupe et al. in the development of the Nijmegen Decision Tool for Chronic Low Back Pain, such a model should be based on substantial amounts of high-quality data, be externally validated, and have a system for continuous monitoring and updates. In practice, the application should incorporate patient-specific information in decision making, show appropriate treatment alternatives with potential benefits and drawbacks, and be delivered on a user-friendly software platform (44). These specifications emphasize the significance of overcoming regulatory and technological hurdles in data collection and storage in order to train population-representative decision algorithms capable of reaching high levels of accuracy in all patients (45, 46).

## Orthopaedic oncology

AI has been investigated in orthopaedic oncology for a variety of applications, including both primary bone or soft tissue tumors and metastatic diseases. While the field is still in its early stages, with few clinical applications, encouraging results have been published in the literature.

### Automated image-based diagnosis

An area of interest for application of AI-based technologies in orthopaedic oncology has been imaging-based diagnosis of tumors. While indolent benign tumors and aggressive cancers are usually evident on plain radiographs, many bone lesions fall into an intermediate category where their histological nature may not be immediately evident on imaging (47). It is the interpretation of these studies that could benefit strongly from AI/ML. The review of intermediate-grade cartilaginous tumors is of particular interest (48).

Even though computer-assisted diagnosis of bone tumors dates back to the 1960s, using non-ML methods, in recent years deep learning based algorithms have been used to classify primary bone tumors as benign or malignant on radiographic images with similar performance compared to clinicians (49). In a multi-institutional study, He et al. used 1,356 radiographs from histologically confirmed primary bone tumors to train a deep learning model (efficientNet-B0 CNN) for differentiating benign, intermediate, and malignant tumors. On external validation using data that was not used for training of the CNN, their model achieved an accuracy of 73.4% compared to average accuracy of 71.3% between two subspecialty trained radiologists (50). In a study with similar design, Eweje et al. used 1,060 T1- and T2-weighted preoperative MRI images to train the same neural network for differentiating benign vs. malignant primary bone tumors. They reported accuracy of 73%, identical to that of radiologists (51). Development of such algorithms could aid in image based diagnosis for intermediate cases, hence reducing the need for invasive diagnostic procedures.

### Clinical outcome prediction

Accurately predicting remaining life expectancy would enhance medical decision-making in orthopaedic oncology, such as helping determine if surgery should be performed and, if so, which surgical treatment should be used (52). Han et al. used deep learning to predict survival rates of patients with synovial sarcoma. Using demographic and clinical data including tumor size, location, initial metastasis, and treatment modality from 242 patients across 3 institutions, a trained neural network model showed improved performance compared to Cox proportional hazard model with AUC of 0.81 compared to 0.63, respectively (53). In a study using the Surveillance, Epidemiology, and End Results database, Ryu et al. investigated the utility of a similar neural network in survival analysis in patients with spinal and pelvic chondrosarcoma. Using data from 1,088 patients authors reported an AUC of 0.84 for predicting survival outcomes. The study did not compare this performance with other non-ML methods of survival analysis (54).

Prediction of local recurrence in primary bone tumors is another area of focus for clinical outcome prediction using AI. Previously, clinical and imaging features have been demonstrated to have utility in predicting probability of local recurrence of bone tumors. For example, factors such as involvement of proximal tibia, younger age, irregular margins or paint brush-border sign, and adjacent soft tissue invasion have been correlated with increased rate of recurrence of giant cell tumor of bone (GCTB) (55, 56). These tumors that are usually managed with intralesional curettage have a recurrence rate of 12%–65%. He et al. used inception v3 CNN on MRI images of 56 patients with GCTB that were followed for an average of 6 years. By combining imaging and patient data (age, and tumor location) they reported an accuracy of 78.6% in correctly predicting recurrence. The clinical application of these findings is not clearly established, however, we believe these forecasts could be used to determine duration and intensity of postoperative surveillance to evaluate for recurrence.

### Segmentation

One of the major applications of AI has been image segmentation. The main benefit from these algorithms is time saving capability, as tasks such as tumor burden analysis or whole body segmentation that had to be done manually with slice-by-slice segmentation now can be performed in fraction of a second. In orthopaedic oncology, image segmentations are utilized to provide a range of quantitative information for clinical decision making, such as neoadjuvant chemoradiation treatment planning and assessment of postoperative therapeutic effectiveness. Most of the research in this area has been focused on CT due to its high contrast and spatial resolution for visualization of bony structures (57).

Zhang et al. used CT images to train a multiple supervised residual network to segment osteosarcoma from 23 patients. The network achieved a dice similarity coefficient, a metric for comparing segmentations to reference, of 89.2% when compared to ground truth segmentations provided by radiologist (58). Lindgren et al. used AI-based automatic segmentation of 18F-sodium fluoride positron emission tomography (PET)/CT images to calculate tumor burden of bone metastasis from prostate cancer. By segmenting the skeleton on CT and areas of high uptake on PET images, authors reported a tumor burden index (Volume_hotspot_/Volume_skeleton_) that was significantly associated with overall survival and correlated with bone scan index (59, 60).

## Trauma

In the current scientific landscape, the applications of AI in orthopaedic trauma are mostly focused on the automated image-based diagnosis of fractures. The research of AI in trauma clinical outcome prediction, although still in its early stages, has also started to emerge.

### Automated image-based diagnosis

The most abundant application of artificial intelligence in trauma orthopaedics described in the literature is the use of deep learning methods, most commonly CNNs, for the detection of fractures. For example, in the highly cited 2017 article by Olczak et al. five deep learning networks were adapted to detect fractures in 256,000 wrist, hand, and ankle radiographs (61). All of these networks accurately identified laterality, body part, exam view, and fracture; the best performing network exhibited a final detection accuracy of 83% for fractures.

When training of deep learning networks from radiographic images is focused on detecting specific types of fractures, higher detection accuracies with significantly reduced sample sizes have been achieved. For example, CNNs exhibited maximum detection accuracies of 94.4%, 95.5%, and 96% for the detection of femoral neck, intertrochanteric hip, and distal radius fractures, respectively (62–64). Other authors have further utilized deep learning methods not only for the detection, but also functional classification of fracture subtypes. These features were highlighted in proximal humeral, calcaneal, and pelvic fractures (65–67).

It is also important to note that when mentioned, the ground truths in these studies were assigned by orthopaedic surgeons, ranging from fourth year postgraduate residents (62) to subspecialists with over a decade of experience (60). Perhaps not surprisingly, all results pointed to comparable, if not better detection accuracies of these networks when compared to expert performance. Lindsey et al. described how a deep neural network successfully improved clinician sensitivity and specificity while detecting fractures in the emergency setting (68). With assistance from the deep neural network, the average clinician's sensitivity and specificity were improved from 80.8% to 91.5% and from 87.5% to 93.9%, respectively. In addition, the misinterpretation rate was reduced by 47%.

Although impressive, the results of these studies are not without limitations. For example, the final accuracies of these deep learning networks seemed to be significantly affected by the quality of the input processing, such as image labeling and cropping, which were done by humans in all of the above studies (60–67); smaller cropped images in Urakawa et al. (62) led to higher diagnostic accuracies of the deep learning networks compared to whole hip radiographs in Olczak et al. (60). Deep learning networks also were not able to perform contextual diagnosis (based on clinical questions posed by ordering clinicians) and prognostication, as they require synthesis of clinical knowledge that cannot be acquired through training sets alone. Furthermore, almost all of the training sets used consisted of images obtained from a single view – findings that only present on one view in multiview radiographs may be missed this way. Regardless of these limitations, it is clear that AI has the potential to transform the landscape of trauma orthopaedics as a diagnostic tool, especially as these limitations are addressed in the near future.

### Clinical outcome prediction

Although the automated detection of fractures still dominates the application of artificial intelligence in trauma orthopaedics, prognostication of patient outcomes is an emerging field of study. Similar to the works of Navarro et al. (18) and Ramkumar et al. (19) in total knee and hip arthroplasty, Karnuta et al. (68) used a naive Bayesian machine-learning algorithm to predict hip fracture patients' length of stay and cost based on patient characteristics including age, ethnicity, gender, and comorbidities. This information was then used in proposing a patient-specific, tiered bundled payment model that balances risk sharing between the payor and institution, which mediates the current challenges in the bundled payment model for hip fracture patients. Although the machine learning algorithm only predicted the most likely payment strata for each patient and not precise value, this study nicely demonstrated the inter-subspecialty applicability of Bayesian machine learning algorithm in predicting orthopaedic patient outcomes, a feature that can even be expanded to other areas of medicine in the near future.

Emerging use of AI in trauma prognostication is also demonstrated by Stojadinovic et al.The authors used a Bayesian classifier to predict the non-union treatment success by extracorporeal shockwave therapy (69, 70). It nicely identified two variables that had the highest predictive values for treatment success (time to therapy and type of bone involved). It is important to note, however, that the use of extracorporeal shockwave therapy is currently restricted to patients with fractures that are refractory to “first line” treatments, such as surgical fixation and cast immobilization. These patients only comprise the minority of fracture patients, and as far as we are aware, AI prognostication studies for fracture patients that underwent surgical or immobilization are still lacking.

In the near future, it would be interesting to track the evolution of AI application in this field of orthopaedics, specifically whether it would involve other imaging modalities beyond radiograph, its expansion into other functional applications and involvement of other treatment modalities.

## Sports medicine

In sports medicine, the applications of AI are still mostly confined to automated image-based diagnosis. MRI is the imaging modality of choice, as soft tissues such as the most likely structures involved in injury. Knee injury is the most active area of research, with anterior cruciate ligament (ACL) and meniscal tear detection being the most common application.

### Automated image-based diagnosis

For the two most common soft tissue injuries of the knee, the anterior cruciate ligament (ACL) and meniscal tear, MRI is the modality of choice for diagnosis. However, diagnosing these injuries on MRI can be challenging given the subtlety of some findings, and the expertise of trained radiologists is almost always required. Thus, the development of automated methods in detecting ACL and meniscal tears is the most active area of research in sports medicine AI.

Štajduhar et al. (2016) developed a semi-automated method for the detection of ACL injury (71). Two machine learning models, random forest and SVM, were used to analyze manually selected rectangular regions of interests involving the ACL area on knee MRIs. The result indicated that the SVM model was best at detecting ACL injuries, with an AUC of 0.894 for any ACL injury and 0.943 for complete ACL rupture.In what seems to be the natural study progression in knee soft tissue injury detection, Bien et al. developed MRNet, a CNN that can detect meniscal and ACL injuries on knee MRI (72). The MRNet exhibited AUC values of 0.937, 0.965, and 0.847 for detecting any abnormalities, ACL tears, and meniscal tears, respectively. The performance of MRNet for detecting soft tissue knee injuries was comparable to that of general unassisted radiologists, although the radiologists achieved higher sensitivity in detecting ACL tears and higher specificity in detecting meniscal tears.

Concordant to this result, other studies have also demonstrated that CNNs are the best deep learning method for fully-automated detection of ACL and meniscal injuries. AUCs as high as 0.971 has been demonstrated for ACL injury detection, while the best performing meniscal tear detection CNN has an AUC of 0.94 (73, 74). This particularly high AUC for meniscal tear detection, however, was only achievable when only normal menisci and those with grade 3 intensity tears were included. More subtle grade 1 and grade 2 meniscal tears were excluded from the study. Therefore, the result from this study should be interpreted with caution, especially for the CNN's ability to detect more subtle meniscal injuries.

In conclusion, the CNNs currently described in the literature exhibit their best performance for ACL tear detection, followed by meniscal tear detection. The availability of CNNs that can detect orthopaedic soft tissue injuries in other body parts is still scarce. However, with the rapid development of automated injury detection on MRI in the past several years, particularly with advancement from more recent CNN models, the same concept will likely extend more widely to detect soft tissue injuries in other body parts, such as the shoulder and ankle.

## Conclusion

Given the heavy reliance on radiological imaging for making orthopaedic diagnoses, it is unsurprising that much of the focus of ML within orthopaedics has focused on automating the interpretation of radiologic imaging. At this point it is well established that ML algorithms can match, or in some cases, exceed the accuracy of trained radiologists or orthopaedic surgeons (6, 9, 10, 15, 33, 39, 49). Yet the question remains, why has ML not been widely adopted in clinical practice for this use? Simply demonstrating an algorithm's accuracy may not be sufficient to drive use of ML, rather, the focus should be demonstrating improvement of real-world outcomes when used in conjunction with clinicians, as demonstrated by Lindsey et al. in improving fracture detection (67). Potentially useful metrics could include reduction in time radiologists spend interpreting images, lower misinterpretation rates, improved patient outcomes, and reduced complication rates.

Other common use cases as profiled in this study focus on integration of large data sets to help predict disease and/or surgical outcomes, especially in a field like orthopaedics that is moving increasingly towards patient-specific care. In contrast to automating radiological diagnosis, analyzing large datasets is not a process that can easily be done by humans. Using ML algorithms offers a clear benefit in terms of processing and interpreting complex, patient-specific data. For these uses, demonstrating a high degree of accuracy and having easy to understand outputs will be critical to get users to buy-in. What remains to be seen is the clinical utility of these predictions, and how clinicians and patients can use the outputs of such algorithms to guide care, two critical metrics to demonstrate in future studies.

Another issue that remains a barrier for the adoption of ML is the “black box”, where ML algorithms are sufficiently complex where their results are not readily interpretable by their human users. Some studies have specifically addressed this concern by specifically showing the key data that lead to the algorithm's output, for example highlighting the key parts of a radiograph that lead to the diagnosis (8). This is a step ML developers can take to demystify the “black box” and build trust and buy-in with the human user.

Acquisition of data remains another large barrier to future use cases and widespread clinical use of ML. Large datasets are often required for the training and validation phases of creating a ML algorithm, which can be difficult to obtain in a single institution study. While sharing data across institutions can increase data availability, this brings its own logistical challenges, including data security, legal/IRB requirements, and data heterogeneity across different electronic health record systems. These barriers to data acquisition and sharing may also make validation of models more difficult. For example, a recent review of studies using AI to evaluate radiographic imaging found that only 6% of the 516 studies demonstrated external validity(Kim et al. 2019). As researchers attempt to translate models from the proof of concept stage, validation and real-world clinical assessments will be necessary to build user trust and gain adoption.

Based on the promising results and wide breadth of use cases in the published literature, it is clear that AI and ML is likely to have a large impact in the future of orthopaedics. As the field of ML progresses, it is likely that improvements to current technologies and development of new applications within orthopaedics will only increase the utility and use of ML in clinical practice. However, ML developers must continue working through the hurdles discussed in this paper to improve trust, familiarity, and ease of use for clinician users and to navigate the ever changing regulatory landscape for AI products. When identifying “best” areas for AI/ML applications, developers should consider two primary features: (1) areas of strong interpretive uncertainty; (2) areas of substantial time and resource consumption. Rationales deriving from the former consideration will apply to clinicians and patients; those deriving from the latter will appeal to clinicians, medical centers, and payers. In addition, other concerns such as medical ethics, data security, and patient privacy will play a critical role in the continued development and use of these technologies. As such, despite the future promise of ML technologies, many steps remain before they can be translated to widespread use in the clinical field of orthopaedics.
